# Dynamic Changes in Amino Acid Release Patterns of Different Plant Protein Sources During In Vitro Digestion and Their Nutritional Value Assessment

**DOI:** 10.3390/ani15213094

**Published:** 2025-10-24

**Authors:** Yueli Fan, Zehua Kou, Jiahua Cao, Zhongshen Wang, Tianrui Zhang, Rui Han, Dongsheng Che

**Affiliations:** 1 Ministry of Education Laboratory of Animal Production and Quality Security, Jilin Agricultural University, Changchun 130118, China; 15774433700@163.com (Y.F.); kouzehua001@163.com (Z.K.); cjh2632704109@163.com (J.C.); 15585538818@163.com (Z.W.); zhangtianrui@jlau.edu.cn (T.Z.); 2 Jilin Provincial Key Laboratory of Animal Nutrition and Feed Science, Jilin Agricultural University, Changchun 130118, China; 3 College of Animal Science and Technology, Jilin Agricultural University, Changchun 130118, China

**Keywords:** protein sources, in vitro digestion, amino acid score, nutritional value equivalents, precision nutrition

## Abstract

**Simple Summary:**

A gastric–intestinal enzymatic hydrolysis digestion method compared five plant protein sources: soybean meal, rapeseed meal, corn DDGS, corn gluten meal, and corn germ meal. Results showed soybean meal’s protein hydrolysis reached 61.8% in the gastric digestion phase, significantly higher than rapeseed meal (43.8%), corn DDGS (31.0%), and corn gluten meal (14.0%). In the intestinal digestion phase, soybean meal’s total nitrogen and low-molecular-weight peptide release were 61.8 mg/g and 42.9 mg/g, higher than those of corn DDGS (17.9 and 17.4 mg/g). A “nutritional value equivalence” model using soybean meal showed rapeseed meal’s gastric digestion phase equivalence was 32.2%, and corn gluten meal’s intestinal phase equivalence was 62.9%. This study provides a basis for soybean meal substitution in feed formulations and precise nutritional requirements.

**Abstract:**

A gastric–intestinal two-step enzymatic hydrolysis in vitro digestion simulation system was used to systematically investigate the digestion kinetics and amino acid release characteristics of five plant protein sources: soybean meal, rapeseed meal, corn DDGS, corn gluten meal, and corn germ meal. The results showed that in the gastric digestion phase (120 min), the protein hydrolysis degree of soybean meal was the highest (61.8%, *p* < 0.001), which was 4.4 times that of corn gluten meal (14.0%). In the intestinal digestion phase (240 min), the low-molecular-weight peptide release of corn gluten meal (31.2 mg/g) was significantly higher than that of corn DDGS (17.4 mg/g), showing a “weak in the stomach but strong in the intestine” characteristic. The “nutritional value equivalence” model constructed with soybean meal as the reference showed that the gastric digestion phase equivalence of rapeseed meal was only 32.2% (significantly lower than other materials), and the intestinal digestion phase equivalence of corn gluten meal was 62.9%. This study clarified the differences in digestion characteristics and key related indicators of different plant protein sources, providing quantitative references and scientific support for the food and feed industries to precisely select protein sources according to digestion phases and optimize the formula design.

## 1. Introduction

The increasing global population and upgrading of dietary structures are driving the continuous rise in demand for high-quality protein [1]. Proteins are not only the structural basis for animal tissues and organs but also participate in the synthesis of enzymes, hormones, and immune-active substances, directly affecting growth, health, and production performance [2]. The digestion efficiency and amino acid release patterns of different plant protein sources in the gastrointestinal tract vary significantly owing to differences in protein structure, amino acid composition, and anti-nutritional factors [3]. The application of current plant protein sources is characterized by significant quality differences and utilization limitations. As a traditional superior protein source, soybean meal has become an industry benchmark owing to its balanced amino acid profile. However, its global supply chain is significantly affected by trade fluctuations. After the implementation of the “Soybean Meal Reduction and Substitution Action” in China’s feed industry in 2024, its usage decreased by 8.0% year-on-year, highlighting the risks associated with dependence on a single protein source [4]. Rapeseed meal, the second-largest protein source, has a digestion rate approximately 30% lower than that of soybean meal due to the presence of anti-nutritional factors such as glucosinolates [5,6]. Corn processing by-products show polarization: corn gluten meal can have a protein purity of over 60%, but it is rich in indigestible zein proteins [7,8]. In contrast, corn DDGS and germ meal, despite their high output, have essential amino acid contents of only approximately one-third of that of soybean meal due to fiber encapsulation and processing denaturation issues. These differences in material characteristics directly lead to significant variations in digestion and absorption efficiencies [9,10]. Anti-nutritional factors, protein structural complexity, and the impact of processing technology collectively form the main barriers to the utilization of plant proteins.

The limitations of existing evaluation systems further restrict the precise application of plant proteins. Traditional methods often rely on static indicators, such as crude protein content or hydrolysis degree, at a single time point, which fail to reflect the dynamic changes in the digestion process [11]. Although the PDCAAS standard promoted by the US FDA considers digestibility correction, it still cannot capture the amino acid release patterns at different digestion stages, leading to cognitive biases in the nutritional value of plant proteins. A more prominent issue is that different studies use a wide variety of detection methods and evaluation indicators, lacking a standardized system for horizontal comparisons [12]. This makes it impossible to scientifically quantify the nutritional value equivalence between soybean meal and other alternative materials, which is in sharp contrast to the feed industry’s demand for precise formulations. Therefore, establishing a dynamic, multi-dimensional evaluation system for plant protein digestion is of great significance for solving the industry dilemma of “abundant resources but low utilization efficiency [13,14].”

This study evaluated the amino acid release kinetics of five by-product proteins in the gastric and intestinal stages using a gastric–intestinal two-step enzymatic hydrolysis in vitro digestion method and constructed a nutritional value equivalence model using soybean meal as the benchmark, providing a scientific basis for the precise utilization and formulation optimization of plant proteins [15,16,17].

## 2. Materials and Methods

### 2.1. Experimental Materials

Experimental Groups: soybean meal, rapeseed meal, corn gluten meal, corn DDGS, and corn germ meal. were purchased from Jiajiakang (Jilin) Co., Ltd. (Changchun, China) in September 2023. A minimum lot of 5 kg per ingredient was sampled using the quartering method to ensure representativeness. Immediately after purchase, samples were ground through a 1 mm screen using a fluidized-bed grinder, vacuum-sealed in polyethylene bags, and stored at 4 °C until analysis. All samples were used within 3 months of collection.

### 2.2. In Vitro Digestive Enzymes

Porcine pepsin (250 U/mg, Sigma, P7000, Co., St. Louis, MO, USA), porcine pancreatin (4 × USP specifications, P1750; Sigma, USA) and porcine bile extract (Sigma, 48305, USA), salivary-α-amylase (30 U/mg, Sigma, 10065, USA), starch glucosidase (120 U/mg, Sigma, 10113, USA).

### 2.3. Experimental Instruments

Laboratory routine instruments, 4 °C ultra-high-speed centrifuge (Eppendorf 5810 R Eppendorf 5810 R, Eppendorf AG, Hamburg, Germany), automatic fiber analyzer (ANKOM 2000, ANKOM Technology, Macedon, NY, USA), automatic Kjeldahl N analyzer (Foss 8400, Foss Analytics A/S, Hillerød, Denmark), muffle furnace (Shanghai Boxun SX2-4-10, Shanghai Boxun Industrial Co., Ltd., Shanghai, China), electric blast drying oven (Shanghai Yiheng, Scientific Instrument Co., Ltd., Shanghai, China), 10,000 points of the electronic analytical balance (Sartorius BSA224S, Sartorius Lab Instruments GmbH & Co. KG, Göttingen, Germany) Multi-functional enzyme labeller (Thermo scientific 1510, Thermo Fisher Scientific Inc., Waltham, MA, USA), Flow mill (Ruian Hao bo BM-103B, Ruian Haobo Machinery Co., Ltd., Wenzhou, Zhejiang, China), Constant temperature air bath shaker (Shanghai Zhi chu ZQTY-50E, Shanghai Zhichu Instrument Co., Ltd., Shanghai, China).

### 2.4. Continuous In Vitro Digestion Simulation Steps

The in vitro digestion kinetics were determined following the methods described by Boisen and Fernández, with some modifications [15,16]. A continuous simulation of in vitro digestion was conducted using individual sample tubes at each time point. For each simulated cultivation in the gastrointestinal digestion of the raw material samples, six replicate tubes were set up as replicate groups (0.3000 ± 0.0003 g of sample was added to each replicate group). Salivary-α-amylase was added to each reaction tube (0.6 mL, 2.5 g/L, pH 7.0), and the samples were incubated in a constant temperature air bath shaker (39 °C) at a constant speed (180 rpm) for 2 min to simulate oral digestion. Following the oral digestion cultivation, pre-warmed phosphate-buffered solution (21 mL, 0.1 M, pH 6.0) and hydrochloric acid solution (0.6 mL, 1 M) were added, and the pH was adjusted to 2.5 with 1 M HCl or 1 M NaOH to simulate the gastric digestion environment. Freshly prepared pepsin solution (0.6 mL, 10 g/L) was added to simulate enzymatic digestion in the stomach. The reaction tubes were sealed and placed in a constant temperature air bath oscillator (39 °C) with continuous stirring (180 rpm) to simulate the stirring and body temperature during digestion. Sampling was conducted at 0, 30, 60, 90, and 120 min. The digestion mixture collected at each time point was rapidly frozen in liquid nitrogen.

To avoid the influence of different sampling times, additional replicate groups were set up for the simulation of the small intestine digestion. The simulation of small intestine digestion was based on the simulated gastric digestion cultivation. The pH was adjusted to 6.8 with 1 M NaOH, followed by the addition of freshly prepared starch glucosidase (0.1 g/mL) and a mixture of porcine pancreatic protease (4.35 mL, 100 g/L) and bile solution (1.5 mL, 150 g/L). The samples were further incubated in a constant temperature air bath shaker (39 °C) with a constant stirring rate (180 rpm) to simulate the digestive environment of the small intestine. Sampling was conducted at 0, 30, 60, 120, 180, and 240 min. The digestion mixture collected at each time point was rapidly frozen in liquid nitrogen.

The samples were centrifuged (15 min, 4000 rpm, 4 °C) to separate the insoluble protein fraction (IPF) and the soluble protein fraction (SPF). Then, 8 mL of the supernatant was separated and mixed with 20% sulfosalicylic acid at a ratio of 1:1 (*v*/*v*) and allowed to precipitate for 30 min. The precipitated samples were centrifuged (10 min, 4000 rpm, 4 °C) to separate the soluble high molecular weight (HMW) peptides and the soluble low-molecular-weight (LMW) peptides [15,16,18]. The supernatant samples after two separations were frozen at −20 °C for subsequent chemical analysis. To calibrate the analysis results, control groups without digestive substrates were added during the gastric and small intestine digestions (six replicates).

### 2.5. Chemical Analysis

All chemical analyses were performed using standard laboratory methods. Crude ash (method GB/T 6438-2007, 2007) [19], ether extract (method GB/T 6433-2025, 2025) [20], moisture (method GB/T 6435-2014, 2015) [21] and crude fiber (method GB/T 6434-2022, 2022) [22] were analysed for the 5 ingredients; Kjeldahl N fixation was used to determine the 11 ingredient samples and their N contents in SPF and LMW supernatants corresponding to different incubation times (methods GB/T 6432-2018, 2019) [23]. Free amino acids (FAAs) were analysed by HPLC at each collection time point [24,25].

### 2.6. Calculation of Digestive Dynamics Parameters

The degree of hydrolysis (DH) is defined as the ratio of the number of peptide bonds, denoted as h1 − h0 (the quantity of free amino acids), cleaved at a given time point during the proteolysis process to the total number of peptide bonds(h_tot_). In this study, the OPA method was employed to measure the absorbance at 340 nm, and the total amount of free amino acids was determined through acid hydrolysis (6 M HCl, 110 °C, 24 h). A standard curve was constructed using a 0.5 mg/mL serine solution.

The formula for calculating DH is as follows (Equation (1)):

(1) $$\mathrm{DH}\left(\%\right)\;=\;\frac{h1\;-\;h0}{h_{\mathrm{tot}}}\;\times \;100\%$$

Herein, h1 represents the concentration of free amino acids in the sample at various time points during the digestion process, h0 denotes the concentration of free amino acids in the sample at the initiation of digestion, and h_tot_ signifies the total concentration of free amino acids. This formula reflects the efficiency of protein hydrolysis into free amino acids under specific digestive conditions and serves as an important metric for assessing the digestive characteristics of proteins.

Calculate N solubility (N_solubility_) using the following equation (Equation (2)) [16]:

(2) $$N_{\mathrm{solubility}}\left(\%\right)\;=\;\frac{N_{\mathrm{sample}}\;-\;(N_{\mathrm{SPF}}\;-\;N_{\mathrm{blank}})}{N_{\mathrm{sample}}}\times \;100\%$$

where N_sample_ (mg) is the amount of N in 1 g of protein source. N_SPF_ (mg) is the amount of N in SPF during sequential incubation with pepsin and porcine pancreatase. N_blank_ (mg) is the amount of N in the SPF of the blank sample during sequential incubation with pepsin and porcine pancreatase.

The N present in the LMW(N_LMW_) is calculated by the following equation (Equation (3)) [16]:

(3) $$N_{\mathrm{LMW}}\;=\;\frac{N_{\mathrm{LMW}}\;-\;N_{\mathrm{blank}}}{N_{\mathrm{sample}}}\times \;100\%$$

where N_LMW_ (mg) is the amount of N in the LMW during sequential incubation with pepsin and porcine pancreatin, N_blank_ (mg) is the amount of N in the LMW of a blank sample during sequential incubation with pepsin and porcine pancreatin, and N_sample_ (mg) is the amount of N in 1 g of protein source.

The digestion kinetics of N_solubility_ and N_LMW_ of different protein sources during incubation were described using the exponential equation (Equation (4)) [26]:

(4) $$D_{t}\;={\;D}_{0}\;+\;∆D\;\times \;(1\;-\;e^{-\mathrm{kt}})$$

where D_t_ (%) is the N of N_solubility_ at incubation time t (min), D_0_ (%) is the N of N_solubility_ at 0 min, ΔD (%) is the maximum N_solubility_ or N corrected by D_0_ (asymptote), and k is the rate constant [26].

### 2.7. Determination of Amino Acid Release and Calculation of Amino Acid Synchronicity

A Waters ACQUITY UPLC I-CLASS system (Waters, Milford, MA, USA) and an ACQUITY UPLC^®^ BEH C18 column (2.1 mm × 100 mm, P/N: 186002352) are utilized according to the manufacturer’s standard protocol with the following parameters: mobile phase A is 1% sodium acetate; mobile phase B is 100% methanol; flow rate is 0.3 mL/min; injection volume is 1 µL; column temperature is 55 °C; sample temperature is 5 °C; excitation wavelength is 340 nm; emission wavelength is 450 nm; collection rate is 20 GS/s; time constant is 0.1 s; and run time is 10 min. Pre-column derivatization of amino acids using o-Phthalaldehyde (OPA) solution with a gradient elution of 14–30% B at 0 min–6 min, 30–70% B at 6 min–12 min, 70–100% B at 12 min–17 min, 100–14% B at 17 min–19 min. The compositions and contents of 17 amino acids are expressed in units of g/100 g protein.

Amino Acid Score (AAS) was applied to evaluate the nutritional value of free amino acids released from raw materials after digestion, this study designed a raw material amino acid score based on the digestibility-corrected amino acid score model (FAO/WHO Expert Consultation, 1991) with minor modifications. The reference protein amino acid content for young children (1–2 years old) and adults (over 18 years old) was derived from the 2007 report of WHO/FAO/UNU [27,28,29,30,31].

The total asynchronicity index of amino acid release was calculated by summing the standard deviations of the percentage release of total amino acids and 17 individual amino acids at different time points for each group [17]. The specific calculation method is shown in Equation (5). A lower asynchronicity index indicates better synchronicity.

(5) $${\mathrm{AA}}^{2}(\%)=\sum_{l}^{11}\sqrt{\frac{1}{17}\sum_{i=\mathrm{aa}}^{17}\left(X_{i}\;-\;\overset{-}{X}\right)}$$

where AA^2^ represents the Total Asynchronicity Index of Amino Acid Release; l represents different digestion time points; i represents different types of amino acids; and $\overset{-}{X}$ represents the average percentage release of amino acid i at all times.

The nutritional value equivalents of other protein sources relative to soybean meal were calculated based on the total amino acid release and the amino acid synchronicity index (Equation (6)):

(6) $$\mathrm{MEQ}\;=\frac{{\mathrm{TAA}}_{\mathrm{sample}}\;\times \;{\mathrm{AA}}_{\mathrm{sample}}^{2}\;\times {\;K}_{\mathrm{sample}}\;\times \;\left(D_{\max}\;-\;D_{0}\right)\mathrm{sample}}{{\mathrm{TAA}}_{\mathrm{soybean}}\;\times \;{\mathrm{AA}}_{\mathrm{soybean}\;}^{2}\times \;K_{\mathrm{soybean}}\;\times \;\left(D_{\max}\;-\;D_{0}\right)\mathrm{soybean}}$$

where MEQ is the nutritional value equivalence based on soybean meal, TAA_soybean_ is the total amino acid release, AA^2^_soybean_ is the synchronicity index of amino acids, TAA_sample_ is the total amino acid release of other samples, and AA^2^_sample_ is the synchronicity index of amino acids.

### 2.8. Statistical Analyses

Data were organized using Excel (Office 2021, Microsoft Corporation, Microsoft Corporation, Redmond, WA, USA). The normality and homogeneity of variance of the data were tested using Levene’s test in SPSS (SPSS 23.0; IBM Corporation, Armonk, NY, USA). A one-way analysis of variance (ANOVA) was performed to examine the differences between the groups. Tukey’s post hoc test confirmed statistically significant differences between the groups (*p* < 0.05). The results are expressed as the mean ± standard error of the mean (SEM). The kinetic equations were fitted using the custom nonlinear fitting program in Origin (Origin 2021, Origin Lab Corporation, Northampton, MA, USA).

## 3. Results

### 3.1. Nutritional Composition and Protein Hydrolysis Degree Characteristics of Different Raw Materials

The proximate compositions of the five plant protein sources are presented in Table 1. Significant differences (*p* < 0.01) were observed in all measured nutritional indices, indicating marked compositional diversity among feed ingredients. Corn gluten meal contained the highest crude protein (CP, 61.17 ± 0.60%) and the lowest crude fiber (CF, 2.59 ± 0.35%), reflecting its high protein purity and low structural carbohydrate content. In contrast, corn germ meal had the lowest CP (19.40 ± 0.41%) but the highest nitrogen-free extract (NFE, 56.75 ± 0.65%), implying greater starch and soluble carbohydrate content.

Soybean meal exhibited a balanced nutrient profile, with relatively high CP (47.23 ± 0.31%) and moderate NFE (31.62 ± 1.02%), making it a representative high-quality protein source. Rapeseed meal showed intermediate CP (38.95 ± 1.65%) but higher CF (11.55 ± 1.05%) and ash (7.71 ± 0.21%), which could potentially reduce digestibility. Corn DDGS displayed the highest ether extract (EE, 8.51 ± 0.67%), indicating high residual oil content from ethanol processing. Collectively, these compositional variations suggest that protein digestibility and amino acid availability would differ markedly among these plant-based ingredients.

As shown in Table 2, during the simulated gastric intestinal continuous digestion process, the protein hydrolysis degree (DH) of different protein sources showed extremely significant differences in both digestion stages (*p* < 0.001). In the gastric digestion stage (120 min), the degree of hydrolysis ranking was as follows: soybean meal (61.84%) > rapeseed meal (43.75%) > corn germ meal (39.51%) > corn DDGS (30.96%) > corn gluten meal (13.99%). Soybean meal exhibited a “fast start—stable and efficient” characteristic, with a hydrolysis degree of 48.32% in the first 30 min, followed by a plateau. In contrast, corn gluten meal exhibited a continuous low-speed growth pattern without a distinct plateau. The digestion processes of the five raw plant protein materials showed significant stage differentiation characteristics. In the intestinal digestion stage (240 min), the hydrolysis degree increased by 10.2–46.8% compared with the gastric stage, and the ranking changed to soybean meal (82.04%) > rapeseed meal (65.31%) > corn gluten meal (44.28%) > corn DDGS (42.36%) > corn germ meal (41.25%). Notably, corn gluten meal showed a significant digestive lag compensation effect in the intestinal stage, with a hydrolysis degree increase of 71.6%, the highest among all raw materials, whereas corn DDGS had the smallest increase (36.8%) and reached a digestion plateau (42.10%) after 180 min, indicating that its protein components are difficult to hydrolyze further.

### 3.2. Nitrogen Release Patterns and Digestion Kinetic Parameters of Different Raw Materials During In Vitro Digestion Process

As shown in Table 3 and Table 4, the total nitrogen release pattern indicates that during the gastric digestion phase (2 h), rapeseed meal and soybean meal had the highest release (both 11.08 mg/g at 120 min), and corn DDGS had the lowest total nitrogen release (9.04 mg/g at 120 min). In the intestinal digestion phase (4 h), the total nitrogen release from soybean meal significantly increased, reaching 61.84 mg/g at 240 min, far exceeding other protein sources; rapeseed meal was the second highest (46.83 mg/g), and corn germ meal had the lowest release (19.45 mg/g). The release pattern of low-molecular-weight peptides (LMW) differed from that of total nitrogen release. During the gastric digestion phase, rapeseed meal had the highest LMW release (22.10 mg/g at 120 min), followed by soybean meal, which had the second highest (10.64 mg/g), and corn DDGS had the lowest release (7.53 mg/g). In the intestinal digestion phase, soybean meal had the highest LMW peptide release (42.85 mg/g at 240 min), followed closely by corn gluten meal performed remarkably well (44.89 mg/g), while corn germ meal had the lowest release (12.93 mg/g). These differences may be related to the peptide chain cleavage patterns and enzymatic sensitivities of different protein sources.

**Figure 1:**
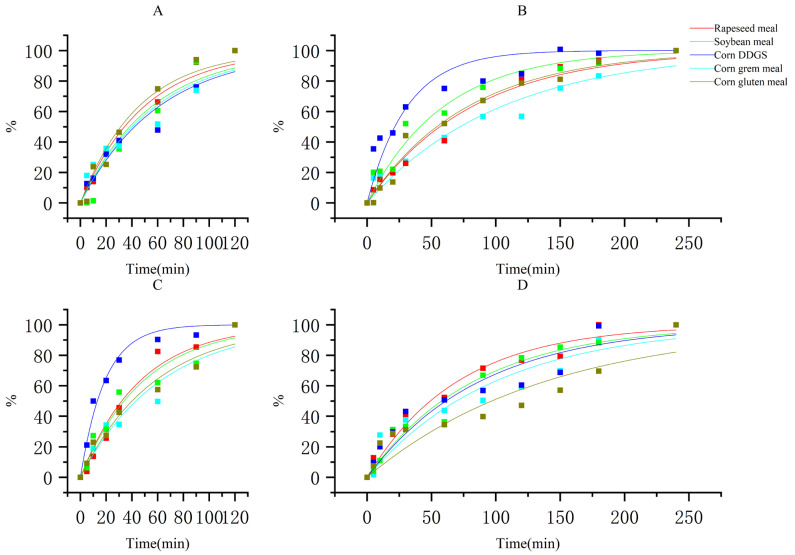
Fitting curves of digestion kinetics models for different protein sources. Note: (**A**) Kinetic curve of nitrogen release during gastric digestion. (**B**) Kinetic curve of nitrogen release during intestinal digestion. (**C**) Kinetic curve of low-molecular-weight peptide release during gastric digestion. (**D**) Kinetic curve of low-molecular-weight peptide release during intestinal digestion.

As shown in Figure 1 and Table 5, the digestion kinetic parameters based on fitting show that rapeseed meal exhibited “fast-digesting” characteristics during the gastric digestion phase. The maximum solubility (28.92%) and rate constant (2.02 min^−1^) of total nitrogen release for rapeseed meal were the best, indicating the highest nitrogen release efficiency in the stomach of the goat. In terms of LMW release, rapeseed meal had the highest maximum solubility (26.92%), whereas corn DDGS had the highest rate constant (4.87 min^−1^); however, its initial solubility was only 0.26%, limiting the overall release efficiency and showing a “fast start but low potential” characteristic. Soybean meal showed “steady and efficient” performance, with all parameters at relatively high levels.

The kinetic characteristics changed significantly during the intestinal digestion phase. Rapeseed meal had a maximum total nitrogen solubility of 90.45%, but its release rate was the slowest (1.23 min^−1^). Corn gluten meal exhibited the fastest release rate (3.22 min^−1^) and moderate maximum solubility (45.32%). Soybean meal maintained a stable maximum solubility (59.87%) and release rate (1.68 min^−1^), with the best overall performance.

### 3.3. Analysis of Amino Acid Release Patterns and Nutritional Value Assessment

**Figure 2:**
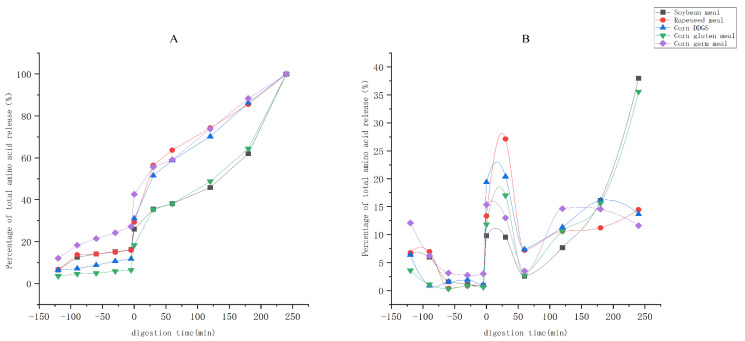
Simulated amino acid release during in vitro digestion of different protein sources. Note: (**A**) represents the cumulative release curves of amino acids during in vitro digestion of different protein sources, and (**B**) represents the incremental release curves of amino acids during in vitro digestion of different protein sources. The period from −120 min to 0 min represents the gastric digestion phase, and the period from 0 min to 240 min represents the intestinal digestion phase.

As shown in Table 6 and Figure 2, during the gastric digestion phase (2 h), soybean meal consistently had the highest amino acid release (6.13 g/100 g at 120 min), corn DDGS had the slowest release growth (only 1.54 g/100 g at 120 min), and rapeseed meal, corn gluten meal, and corn germ meal had releases of 3.52 g/100 g, 1.75 g/100 g, and 3.02 g/100 g, respectively. After the intestinal digestion phase (4 h), the amino acid release of all protein sources significantly increased, with soybean meal reaching 37.86 g/100 g at 240 min, corn gluten meal being the second highest (26.79 g/100 g), rapeseed meal at 22.04 g/100 g, and corn DDGS and corn germ meal having the lowest releases (11.68 g/100 g and 11.08 g/100 g, respectively).

As shown in Table 7 and Table 8, the determination of total essential amino acids indicated that soybean meal had the highest content (226.98 mg/g), followed by corn gluten meal (152.60 mg/g), rapeseed meal (119.09 mg/g), and corn DDGS and corn germ meal had lower contents (68.19 mg/g and 60.45 mg/g, respectively). The amino acid scoring results showed that soybean meal had the highest total score (0.657), with phenylalanine + tyrosine scoring 1.000 (the highest possible score) and leucine having a relatively high score (0.731). Corn gluten meal had a total score of 0.442, with leucine (0.609) and phenylalanine + tyrosine (0.929) being the most prominent. Rapeseed meal had a total score of 0.345, with all amino acid scores lower than those of soybean meal. Corn DDGS (0.198) and corn germ meal (0.175) had the lowest total scores, with most essential amino acid scores below 0.3, indicating a less balanced amino acid composition.

As shown in Table 9, taking soybean meal as the benchmark (nutritional value equivalence 100%), during the gastric digestion phase, rapeseed meal had the highest equivalence (32.17%), while corn DDGS had the lowest (6.69%); during the intestinal digestion phase, the equivalence of rapeseed meal peaked at 99.45% at 60 min, then gradually decreased to 29.52% at 240 min; the equivalence of corn gluten meal peaked at 62.87% at 120 min; corn germ meal consistently had the lowest equivalence (only 7.82% at 240 min). These results provide a quantitative basis for selecting application scenarios for different protein sources.

## 4. Discussion

### 4.1. The Impact of Nutritional Composition on Digestive Characteristics

The differences in the nutritional composition of the five plant protein sources directly affected their digestive performance. Although corn gluten meal has the highest crude protein content, its main component is zein [32]. The core mechanism lies in the fact that the Cys155 residue in zein forms a three-dimensional network structure through disulfide bonds [33,34], which hinders the contact between pepsin and peptide bonds, resulting in a protein hydrolysis degree (DH) in the gastric digestion phase that is only 22.6% of that of soybean meal (*p* < 0.05). Meanwhile, the complex of protein and starch in corn gluten meal further reduces the enzymatic hydrolysis efficiency, which is consistent with the conclusion observed by Li et al. [35,36] that “the interaction between corn protein and starch inhibits the activity of digestive enzymes.”

The crude fiber (11.55%) in rapeseed meal forms a physical barrier in the intestinal phase, enveloping undigested protein particles and reducing the action sites of trypsin [37,38]. Moreover, the isothiocyanates generated from the metabolism of glucosinolates in rapeseed meal can reduce hydrolysis efficiency by inhibiting the serine active sites of trypsin, leading to a 36.6% decrease in low-molecular-weight (LMW) peptide release in the intestinal phase compared with the gastric phase (*p* < 0.05) [39]. The high crude fat content (8.51%) in corn DDGS forms a lipoprotein complex with protein [40]. This complex reduces the binding affinity of pepsin by altering the surface charge of the protein, resulting in a total nitrogen release that is consistently significantly lower than that of soybean meal (*p* < 0.05) [41,42]. Corn germ meal had the lowest crude protein content and high crude fiber content, and these two factors together led to the worst overall digestion efficiency [43].

In contrast, the protein in soybean meal mainly comprises 7S β-conglycinin and 11S glycinin, both of which are prone to dissociate into subunits in the acidic gastric environment, exposing more peptide bonds [44,45]. Moreover, soybean meal has a low content of anti-nutritional factors (such as soybean lectin), which only weakly inhibit digestive enzyme activity. Therefore, it maintains high DH and nitrogen release in both digestion phases, demonstrating its structural advantage as a high-quality plant protein source [46,47].

### 4.2. The Correlation Between Digestive Phase Differences and Protein Source Characteristics

The gastric digestion phase, as the key initial step for protein breakdown, showed the highest degree of hydrolysis for soybean meal, indicating that its protein structure is readily disrupted by pepsin, exposing peptide bonds sufficiently and laying a solid foundation for subsequent intestinal digestion [14,48]. The high initial hydrolysis degree of corn germ meal may be due to its smaller protein molecules or looser structure, which are easily and quickly broken down by gastric acids. However, the limited increase in hydrolysis degree thereafter, influenced by crude fiber, reflects a digestion characteristic of “fast initially but slow later on.” This phenomenon is consistent with the conclusion that the initial structural state of proteins significantly affects their digestive kinetics. The processing methods of plant proteins can alter their intermolecular interactions, thereby influencing their digestive behavior.

The intestinal digestion phase, as the core stage for amino acid absorption, showed that soybean meal had the highest total amino acid release and stable LMW release, indicating that its proteins can be continuously broken down into absorbable small peptides and free amino acids under the action of trypsin [12,49,50]. This is in line with its positioning as a “high-quality plant protein source.” The near-soybean-meal LMW release of corn gluten meal in the intestinal phase may be due to the gradual breakdown of proteins that are not fully decomposed in the gastric digestion phase by trypsin in the intestine. Moreover, its branched-chain amino acids (such as leucine) are easily broken down into smaller peptides [5,13,51]. Therefore, it exhibits a “slow in the stomach but fast in the intestine” digestive characteristic, providing a theoretical basis for its use as an auxiliary protein source to supplement amino acids during the intestinal digestion phase.

### 4.3. Nutritional Value Assessment and Implications for Practical Application

Amino acid scoring further validates the high quality of soybean meal: its amino acid composition is closer to the FAO/WHO recommended pattern. Therefore, establishing a “nutritional value equivalent” model based on essential amino acid score with soybean meal as the benchmark can better demonstrate the application potential of soybean meal substitutes [52]. Rapeseed meal performs well in the gastric phase and can be used in scenarios that require rapid nitrogen and small peptide supplementation; however, it needs to be pre-treated (such as detoxification and enzymatic hydrolysis) to reduce the impact of anti-nutritional factors [53,54]. Studies have shown that pretreatment techniques, such as fermentation, can effectively degrade anti-nutritional factors in by-products, improving amino acid utilization and protein digestibility [55,56].

Corn gluten meal is notable for its low-molecular-weight (LMW) peptide release during the intestinal phase, making it suitable for functional foods that require sustained amino acid release [57]. Corn DDGS and corn germ meal have limited value when used alone as protein sources, but they can be components of composite proteins: corn DDGS is rich in vitamins and minerals and can be combined with soybean meal to enrich nutrition; the dietary fiber in corn germ meal can play an intestinal regulatory role and can be paired with high-protein sources to achieve a dual effect of “nutrition + function [52,53,58].” This application strategy is in line with the principle of protein complementarity; that is, by scientifically adjusting the ratio of different plant protein sources, the amino acid spectrum can be optimized and nutritional balance can be achieved [59,60].

The differences in the digestion kinetic parameters of different protein sources suggest that the ratio of protein sources can be adjusted during food processing to optimize the synchronicity and sustainability of amino acid release [16,61]. For example, a certain proportion of soybean meal and corn gluten meal can be mixed to balance the rapid release in the gastric phase and the sustained supply in the intestinal phase, thereby enhancing the overall nutritional value of the product. Future research should focus on the regulation of protein structure using processing techniques such as extrusion or enzymatic treatment [62,63,64]. The digestibility of plant proteins can be further improved by breaking the disulfide bonds in corn zein and reducing the crude fiber content in rapeseed meal. Simultaneously, in vivo digestion experiments can be combined to verify the reliability of in vitro models, providing a more comprehensive theoretical basis for the precise application of protein source feeds [65,66], as summarized in Table 10.

### 4.4. Translation of In Vitro Findings to In Vivo Performance

Although this study primarily focused on in vitro digestion, the observed amino acid release dynamics among different plant protein sources provide valuable implications for in vivo utilization in target animals [67]. Proteins exhibiting faster hydrolysis rates and more balanced amino acid release profiles in vitro—such as soybean and rapeseed proteins—tend to correspond to greater postprandial amino acid availability and improved nitrogen retention under in vivo conditions [68]. Conversely, proteins with compact molecular structures or residual anti-nutritional factors, such as corn gluten meal, often display slower digestion kinetics and reduced amino acid absorption efficiency [48]. Several studies have reported significant correlations between in vitro digestion kinetics and in vivo measures such as true ileal digestibility, nitrogen balance, and growth performance in pigs and poultry [16,17,61]. Therefore, the dynamic release patterns observed in this work may serve as predictive indicators of in vivo protein utilization efficiency. Future validation using controlled in vivo trials will be valuable to confirm these relationships.

## 5. Conclusions

This study demonstrates that in vitro simulated digestion experiments can effectively evaluate the digestive characteristics and nutritional values of different raw protein materials. Although rapeseed meal has a higher digestion rate in the early stages than soybean meal, its overall digestion potential is limited. Its application value depends on a rational combination with other protein raw materials or improvement of the utilization rate through appropriate pre-treatment. Corn germ meal has relatively low amino acid release and nutritional value equivalence. In contrast, corn DDGS and corn gluten meal show certain substitution potential, providing possibilities for the diversified utilization of protein raw materials. This study not only provides a theoretical basis for the scientific selection, rational combination, and processing optimization of protein raw materials in the feed industry but also offers new ideas for methodological research on the rapid screening and evaluation of protein raw materials. By avoiding the limitations of in vivo experiments, it provides more comprehensive theoretical support for the precise application of protein source feeds.

## Figures and Tables

**Table 1:** Nutritional Composition of Different Foods.

	M (%)	CP (%)	CF (%)	EE (%)	Ash (%)	NFE (%)
Soybean meal	8.42 ± 0.79 ^bc^	47.23 ± 0.31 ^b^	5.39 ± 0.57 ^c^	1.15 ± 0.41 ^c^	6.18 ± 0.23 ^b^	31.62 ± 1.02 ^c^
Rapeseed meal	8.46 ± 1.46 ^bc^	38.95 ± 1.65 ^c^	11.55 ± 1.05 ^a^	1.95 ± 0.35 ^bc^	7.71 ± 0.21 ^a^	31.37 ± 0.85 ^c^
Corn DDGS	11.46 ± 0.84 ^a^	27.75 ± 0.57 ^d^	5.85 ± 0.65 ^c^	8.51 ± 0.67 ^a^	4.54 ± 0.30 ^c^	41.88 ± 2.13 ^b^
Corn gluten meal	7.26 ± 0.53 ^c^	61.17 ± 0.60 ^a^	2.59 ± 0.35 ^d^	2.71 ± 0.24 ^b^	2.04 ± 0.07 ^e^	24.23 ± 0.22 ^d^
Corn germ meal	9.78 ± 0.33 ^ab^	19.40 ± 0.41 ^e^	9.29 ± 0.22 ^b^	2.01 ± 0.30 ^bc^	2.78 ± 0.05 ^d^	56.75 ± 0.65 ^a^
SEM	0.429	3.914	0.850	0.719	0.563	3.028
*p*-value	0.002	<0.001	<0.001	<0.001	<0.001	<0.001

Note: Values are expressed as mean ± SD (*n* = 6). Within a row (fixed time point), different lowercase superscripts indicate significant differences among ingredients (*p* < 0.05, Tukey HSD). The rightmost column shows the one-way ANOVA *p*-value for the ingredient effect at that time point (three decimals or “<0.001”). Abbreviations: M, moisture; CP, crude protein; CF, crude fiber; EE, ether extract; Ash, ash; NFE, nitrogen-free extract.

**Table 2:** In Vitro Protein Hydrolysis Degree of Different Foods.

Item	t/min	Soybean Meal	Rapeseed Meal	Corn DDGS	Corn Gluten Meal	Corn Germ Meal	SEM	*p*-Value
Gastric digestion (2 h)	0	4.56 ± 0.63 ^c^	8.16 ± 1.07 ^b^	7.89 ± 0.81 ^b^	1.73 ± 0.09 ^d^	16.90 ± 1.62 ^a^	1.378	<0.001
	30	14.32 ± 3.29 ^b^	10.49 ± 1.02 ^bc^	11.02 ± 2.12 ^bc^	7.65 ± 0.96 ^c^	21.21 ± 0.88 ^a^	1.311	<0.001
	60	15.80 ± 1.53 ^b^	12.42 ± 0.93 ^bc^	11.57 ± 2.59 ^bc^	9.07 ± 1.13 ^c^	21.91 ± 2.32 ^a^	1.249	<0.001
	90	22.53 ± 1.94 ^b^	14.17 ± 1.21 ^c^	15.25 ± 2.59 ^c^	12.73 ± 1.84 ^c^	27.90 ± 1.54 ^a^	1.603	<0.001
	120	61.84 ± 0.57 ^a^	43.75 ± 1.23 ^b^	30.96 ± 5.37 ^c^	13.99 ± 2.89 ^d^	39.51 ± 2.66 ^b^	4.241	<0.001
Intestinal digestion (4 h)	0	70.86 ± 0.78 ^a^	49.13 ± 1.23 ^b^	38.55 ± 5.11 ^c^	25.52 ± 4.09 ^d^	45.30 ± 1.56 ^bc^	4.028	<0.001
	30	73.99 ± 0.82 ^a^	49.35 ± 1.44 ^b^	39.42 ± 1.65 ^c^	27.52 ± 5.33 ^d^	47.48 ± 2.75 ^b^	4.139	<0.001
	60	76.78 ± 2.73 ^a^	51.70 ± 1.25 ^b^	40.32 ± 3.16 ^c^	29.96 ± 3.39 ^d^	50.86 ± 1.45 ^b^	4.211	<0.001
	120	80.32 ± 0.95 ^a^	52.62 ± 1.47 ^b^	41.05 ± 4.39 ^c^	31.34 ± 3.10 ^d^	50.79 ± 1.71 ^b^	4.429	<0.001
	180	80.68 ± 0.87 ^a^	53.81 ± 4.88 ^b^	42.16 ± 1.16 ^c^	40.64 ± 1.54 ^c^	55.63 ± 1.86 ^b^	3.878	<0.001
	240	82.04 ± 0.53 ^a^	57.83 ± 3.71 ^b^	42.36 ± 0.53 ^c^	44.28 ± 1.89 ^c^	58.74 ± 1.79 ^b^	3.822	<0.001

Note: Values are expressed as mean ± SD (*n* = 6). Within a row (fixed time point), different lowercase superscripts indicate significant differences among ingredients (*p* < 0.05, Tukey HSD). The rightmost column shows the one-way ANOVA *p*-value for the ingredient effect at that time point (three decimals or “<0.001”).

**Table 3:** Solubilization changes in N during in vitro simulated gastric and small intestinal digestion.

Item	t/min	Soybean Meal	Rapeseed Meal	Corn DDGS	Corn Gluten Meal	Corn Germ Meal	SEM	*p*-Value
N Gastric digestion (2 h)	0	4.50 ± 0.86 ^c^	17.01 ± 0.44 ^a^	4.82 ± 0.44 ^c^	7.51 ± 0.94 ^b^	8.28 ± 0.98 ^b^	0.852	<0.001
	30	6.83 ± 1.23 ^cd^	20.88 ± 0.52 ^a^	6.55 ± 1.15 ^d^	11.79 ± 0.74 ^b^	10.04 ± 0.57 ^c^	0.981	<0.001
	60	8.48 ± 1.05 ^cd^	23.51 ± 0.32 ^a^	6.84 ± 1.40 ^d^	13.70 ± 1.53 ^b^	10.80 ± 2.14 ^c^	1.120	<0.001
	90	10.59 ± 0.80 ^d^	26.07 ± 0.73 ^a^	8.03 ± 0.86 ^d^	15.67 ± 1.10 ^b^	11.98 ± 1.10 ^c^	1.181	<0.001
	120	11.08 ± 1.48 ^c^	26.83 ± 0.38 ^a^	9.04 ± 1.40 ^d^	15.93 ± 1.48 ^b^	13.37 ± 1.31 ^c^	1.177	<0.001
N Intestinal digestion (4 h)	0	37.36 ± 0.32 ^b^	30.16 ± 0.67 ^a^	12.80 ± 2.04 ^d^	35.93 ± 1.20 ^b^	13.36 ± 1.71 ^c^	2.012	<0.001
	30	50.11 ± 0.99 ^a^	34.50 ± 1.18 ^a^	16.00 ± 1.94 ^b^	41.68 ± 1.50 ^a^	15.02 ± 0.91 ^b^	2.597	<0.001
	60	51.79 ± 2.33 ^a^	36.96 ± 1.80 ^a^	16.62 ± 0.62 ^b^	42.14 ± 2.39 ^a^	15.97 ± 1.77 ^b^	2.659	<0.001
	120	58.19 ± 2.04 ^a^	43.67 ± 0.59 ^b^	17.11 ± 1.66 ^c^	46.21 ± 1.69 ^b^	16.82 ± 0.92 ^c^	3.095	<0.001
	180	59.87 ± 2.04 ^a^	45.79 ± 1.32 ^b^	17.80 ± 0.44 ^c^	48.09 ± 1.70 ^b^	18.44 ± 1.09 ^d^	3.151	<0.001
	240	61.84 ± 2.44 ^b^	46.83 ± 1.40 ^c^	17.89 ± 0.21 ^d^	49.11 ± 1.64 ^a^	19.45 ± 1.08 ^e^	3.241	<0.001

Note: Values are expressed as mean ± SD (*n* = 6). Within a row (fixed time point), different lowercase superscripts indicate significant differences among ingredients (*p* < 0.05, Tukey HSD). The rightmost column shows the one-way ANOVA *p*-value for the ingredient effect at that time point (three decimals or “<0.001”).

**Table 4:** Solubilization changes in LMW during in vitro simulated gastric and small intestinal digestion.

Item	t/min	Soybean Meal	Rapeseed Meal	Corn DDGS	Corn Gluten Meal	Corn Germ Meal	SEM	*p*-Value
LMW Gastric digestion (2 h)	0	1.42 ± 0.33 ^c^	15.07 ± 1.34 ^a^	0.16 ± 0.03 ^c^	4.35 ± 0.78 ^b^	5.23 ± 0.97 ^b^	0.986	<0.001
	30	6.56 ± 1.23 ^d^	18.27 ± 0.77 ^a^	5.83 ± 0.73 ^d^	9.45 ± 0.59 ^b^	7.42 ± 0.47 ^c^	0.855	<0.001
	60	7.15 ± 0.31 ^cd^	20.87 ± 1.43 ^a^	6.82 ± 0.40 ^d^	11.07 ± 0.67 ^b^	8.42 ± 0.70 ^c^	0.979	<0.001
	90	8.31 ± 0.53 ^c^	21.08 ± 0.55 ^a^	7.03 ± 0.65 ^d^	12.58 ± 1.02 ^b^	10.05 ± 1.36 ^c^	0.939	<0.001
	120	10.64 ± 0.53 ^d^	22.10 ± 0.41 ^a^	7.53 ± 0.30 ^d^	15.65 ± 1.11 ^b^	11.79 ± 2.22 ^c^	0.950	<0.001
LMW Intestinal digestion (4 h)	0	19.10 ± 1.57 ^a^	21.91 ± 0.86 ^b^	7.22 ± 0.54 ^c^	18.93 ± 1.39 ^a^	10.11 ± 0.60 ^c^	1.077	<0.001
	30	27.05 ± 0.92 ^a^	27.43 ± 0.88 ^c^	11.62 ± 1.31 ^d^	27.22 ± 1.02 ^b^	11.17 ± 0.41 ^d^	1.450	<0.001
	60	27.73 ± 1.11 ^a^	29.16 ± 0.29 ^c^	12.40 ± 1.01 ^d^	27.98 ± 1.04 ^b^	11.34 ± 0.77 ^d^	1.505	<0.001
	120	37.71 ± 1.63 ^a^	32.55 ± 1.87 ^c^	13.39 ± 1.01 ^d^	31.07 ± 1.20 ^b^	11.78 ± 0.70 ^d^	1.987	<0.001
	180	40.04 ± 1.97 ^a^	35.78 ± 0.45 ^b^	17.36 ± 1.12 ^c^	36.78 ± 1.27 ^b^	12.65 ± 0.72 ^c^	2.095	<0.001
	240	42.85 ± 1.187 ^a^	35.78 ± 0.74 ^b^	17.43 ± 1.04 ^c^	44.89 ± 0.84 ^b^	12.93 ± 0.93 ^c^	2.450	<0.001

Note: Values are expressed as mean ± SD (n = 6). Within a row (fixed time point), different lowercase superscripts indicate significant differences among ingredients (*p* < 0.05, Tukey HSD). The rightmost column shows the one-way ANOVA *p*-value for the ingredient effect at that time point (three decimals or “<0.001”).

**Table 5:** In Vitro Digestion Kinetics Fitting Parameters of Total Nitrogen for Different Foods.

	Item	Parameter	Soybean Meal	Rapeseed Meal	Corn DDGS	Corn Gluten Meal	Corn Germ Meal	*p*-Value
Gastric digestion (2 h)	Nsolubility	D_0_ (%)	8.62 ± 0.57 ^b^	6.68 ± 0.42 ^bc^	8.02 ± 0.68 ^b^	1.86 ± 0.23 ^d^	17.29 ± 1.12 ^a^	<0.001
		ΔD (%)	6.09 ± 0.38 ^c^	22.24 ± 0.75 ^a^	7.00 ± 0.43 ^c^	2.97 ± 0.20 ^c^	11.42 ± 0.59 ^b^	<0.001
		D_max_ (%)	14.71 ± 0.82 ^b^	28.92 ± 0.94 ^a^	15.02 ± 0.90 ^b^	4.83 ± 0.30 ^c^	28.71 ± 1.01 ^a^	<0.001
		k (min^−1^)	1.81 ± 0.09 ^b^	2.02 ± 0.11 ^b^	0.53 ± 0.05 ^c^	1.18 ± 0.08 ^b^	0.41 ± 0.03 ^c^	<0.001
		R^2^	0.94 ± 0.02	0.98 ± 0.01	0.94 ± 0.02	0.97 ± 0.01	0.92 ± 0.03	–
Intestinal digestion (4 h)	Nsolubility	D_0_ (%)	44.07 ± 1.76 ^b^	63.25 ± 2.04 ^a^	30.41 ± 1.22 ^c^	15.38 ± 0.97 ^d^	40.99 ± 1.61 ^b^	<0.001
		ΔD (%)	15.80 ± 0.90 ^b^	27.19 ± 1.02 ^a^	12.08 ± 0.73 ^bc^	29.94 ± 1.31 ^a^	18.70 ± 0.95 ^b^	<0.001
		D_max_ (%)	59.87 ± 1.35 ^b^	90.45 ± 2.31 ^a^	42.49 ± 1.10 ^c^	45.32 ± 1.12 ^c^	59.69 ± 1.48 ^b^	<0.001
		k (min^−1^)	1.68 ± 0.08 ^b^	1.23 ± 0.06 ^c^	0.10 ± 0.01ᵉ	3.22 ± 0.13 ^a^	2.30 ± 0.11 ^ab^	<0.001
		R^2^	0.97 ± 0.01	0.98 ± 0.01	0.89 ± 0.03	0.97 ± 0.01	0.95 ± 0.02	–
Gastric digestion (2 h)	LMW	D_0_ (%)	6.74 ± 0.45 ^bc^	3.34 ± 0.27 ^c^	0.26 ± 0.03 ^d^	0.75 ± 0.08 ^d^	10.92 ± 0.61 ^a^	<0.001
		ΔD (%)	3.43 ± 0.28 ^c^	23.58 ± 0.95 ^a^	12.25 ± 0.64 ^b^	3.90 ± 0.31 ^c^	14.41 ± 0.70 ^b^	<0.001
		D_max_ (%)	10.17 ± 0.54 ^b^	26.92 ± 1.03 ^a^	12.51 ± 0.68 ^b^	4.65 ± 0.26 ^c^	25.33 ± 0.91 ^a^	<0.001
		k (min^−1^)	2.04 ± 0.10 ^b^	2.16 ± 0.11 ^b^	4.87 ± 0.19 ^a^	1.21 ± 0.07 ^c^	0.66 ± 0.04 ^c^	<0.001
		R^2^	0.95 ± 0.02	0.97 ± 0.01	0.98 ± 0.01	0.96 ± 0.01	0.94 ± 0.02	–
Intestinal digestion (4 h)	LMW	D_0_ (%)	20.68 ± 0.83 ^b^	24.00 ± 0.91 ^a^	17.16 ± 0.72 ^c^	7.29 ± 0.45 ^d^	31.03 ± 1.12 ^a^	<0.001
		ΔD (%)	20.18 ± 0.88 ^b^	42.02 ± 1.35 ^a^	24.24 ± 0.96 ^b^	32.24 ± 1.12 ^a^	8.64 ± 0.54 ^c^	<0.001
		D_max_ (%)	40.85 ± 1.31 ^b^	66.03 ± 1.82 ^a^	41.40 ± 1.33 ^b^	39.53 ± 1.25 ^b^	39.67 ± 1.26 ^b^	<0.001
		k (min^−1^)	1.19 ± 0.06 ^bc^	1.41 ± 0.07 ^b^	4.28 ± 0.18 ^a^	3.99 ± 0.17 ^a^	1.24 ± 0.07 ^b^	<0.001
		R^2^	0.97 ± 0.01	0.97 ± 0.01	0.91 ± 0.02	0.85 ± 0.03	0.90 ± 0.02	–

Note: Values are expressed as mean ± SD (*n* = 6). Different lowercase superscripts within a row denote significant differences among ingredients (*p* < 0.05, Tukey HSD). The *p*-value column shows the ANOVA result for ingredient effect under each condition (three decimals or “<0.001”). R^2^ values describe goodness-of-fit and were not included in the ANOVA.

**Table 6:** In Vitro Digestion Amino Acid Release of Different Protein Sources.

Total Amino Acid Release (g/100 g)	t/min	Soybean Meal	Rapeseed Meal	Corn DDGS	Corn Gluten Meal	Corn Germ Meal	*p*-Value
Gastric digestion (2 h)	0	2.47 ± 0.12 ^b^	1.49 ± 0.09 ^c^	0.83 ± 0.05 ^d^	0.96 ± 0.06 ^d^	1.34 ± 0.08 ^c^	<0.001
	30	4.75 ± 0.21 ^b^	3.03 ± 0.15 ^c^	0.94 ± 0.07 ^d^	1.25 ± 0.08 ^d^	2.03 ± 0.12 ^c^	<0.001
	60	5.36 ± 0.24 ^b^	3.12 ± 0.17 ^c^	1.15 ± 0.08 ^d^	1.34 ± 0.09 ^d^	2.38 ± 0.14 ^c^	<0.001
	90	5.77 ± 0.28 ^b^	3.30 ± 0.18 ^c^	1.40 ± 0.09 ^d^	1.58 ± 0.10 ^cd^	2.69 ± 0.15 ^c^	<0.001
	120	6.13 ± 0.30 ^b^	3.52 ± 0.20 ^c^	1.54 ± 0.10 ^d^	1.75 ± 0.11 ^cd^	3.02 ± 0.18 ^c^	<0.001
Intestinal digestion (4 h)	0	9.85 ± 0.45 ^b^	6.46 ± 0.31 ^c^	4.07 ± 0.25 ^d^	4.93 ± 0.27 ^d^	4.72 ± 0.26 ^d^	<0.001
	30	13.47 ± 0.61 ^b^	12.44 ± 0.55 ^b^	6.74 ± 0.33 ^c^	9.49 ± 0.43 ^c^	6.17 ± 0.31 ^c^	<0.001
	60	14.45 ± 0.66 ^b^	14.03 ± 0.64 ^b^	7.70 ± 0.38 ^c^	10.22 ± 0.49 ^c^	6.55 ± 0.33 ^c^	<0.001
	120	17.36 ± 0.80 ^b^	16.37 ± 0.75 ^b^	9.18 ± 0.46 ^c^	13.07 ± 0.63 ^c^	8.18 ± 0.41 ^c^	<0.001
	180	23.47 ± 1.04 ^b^	18.85 ± 0.94 ^c^	12.68 ± 0.63 ^d^	17.26 ± 0.86 ^c^	9.80 ± 0.49 ^d^	<0.001
	240	37.86 ± 1.65 ^a^	22.04 ± 1.05 ^b^	11.68 ± 0.59 ^c^	26.79 ± 1.28 ^b^	11.08 ± 0.57 ^c^	<0.001

Note: Values are expressed as mean ± SD (*n* = 6). Within a row (fixed time point), different lowercase superscripts indicate significant differences among ingredients (*p* < 0.05, Tukey HSD). The rightmost column shows the one-way ANOVA *p*-value for the ingredient effect at that time point (three decimals or “<0.001”).

**Table 7:** Comparison and analysis of the composition and content of essential amino acids in different plant protein sources.

mg/g	Soybean Meal	Rapeseed Meal	Corn DDGS	Corn Gluten Meal	Corn Germ Meal	FAO/WHO Pattern	*p*-Value
Thr	17.89 ± 0.51 ^a^	9.56 ± 1.28 ^d^	9.23 ± 0.64 ^e^	8.22 ± 0.43 ^b^	4.51 ± 0.94 ^c^	40	<0.001
Val	12.77 ± 0.32 ^a^	6.57 ± 0.44 ^b^	4.14 ± 0.21 ^c^	7.63 ± 0.41 ^b^	4.41 ± 0.49 ^c^	50	<0.001
Met + Cys	23.09 ± 0.41 ^a^	9.80 ± 0.78 ^c^	8.02 ± 0.13 ^d^	14.98 ± 0.82 ^b^	5.26 ± 0.81 ^d^	35	<0.001
Ile	10.33 ± 0.61 ^a^	5.83 ± 0.45 ^b^	3.42 ± 1.03 ^c^	6.92 ± 0.31 ^b^	2.90 ± 0.89 ^c^	40	<0.001
Leu	51.23 ± 1.34 ^a^	32.16 ± 0.39 ^c^	12.73 ± 0.37 ^d^	42.66 ± 0.45 ^b^	8.24 ± 0.98 ^d^	70	<0.001
Phe + Tyr	78.68 ± 1.77 ^a^	38.44 ± 0.58 ^c^	15.27 ± 1.67 ^d^	55.74 ± 0.91 ^b^	26.32 ± 0.75 ^d^	60	<0.001
Lys	32.98 ± 0.54 ^a^	16.73 ± 0.37 ^b^	15.37 ± 0.45 ^b^	16.45 ± 0.62 ^b^	8.81 ± 0.69 ^c^	50	<0.001
Total	226.98 ± 1.89 ^a^	119.09 ± 1.49 ^c^	68.19 ± 2.28 ^d^	152.60 ± 1.26 ^b^	60.45 ± 2.94 ^d^	345	<0.001

Note: Values are expressed as mean ± SD (*n* = 6). Within a row (fixed time point), different lowercase superscripts indicate significant differences among ingredients (*p* < 0.05, Tukey HSD). The rightmost column shows the one-way ANOVA *p*-value for the ingredient effect at that time point (three decimals or “<0.001”).

**Table 8:** Amino acid scores of essential amino acids in different plant protein sources.

AAS	Soybean Meal	Rapeseed Meal	Corn DDGS	Corn Gluten Meal	Corn Germ Meal	*p*-Value
Thr	0.45 ± 0.01 ^a^	0.24 ± 0.03 ^c^	0.23 ± 0.02 ^c^	0.21 ± 0.01 ^c^	0.11 ± 0.02 ^d^	<0.001
Val	0.26 ± 0.01 ^a^	0.13 ± 0.01 ^b^	0.08 ± 0.00 ^c^	0.15 ± 0.01 ^b^	0.09 ± 0.01 ^c^	<0.001
Met + Cys	0.66 ± 0.01 ^a^	0.28 ± 0.02 ^c^	0.23 ± 0.00 ^c^	0.43 ± 0.02 ^b^	0.15 ± 0.02 ^d^	<0.001
Ile	0.26 ± 0.02 ^a^	0.15 ± 0.01 ^b^	0.09 ± 0.03 ^c^	0.17 ± 0.01 ^b^	0.07 ± 0.02 ^c^	<0.001
Leu	0.73 ± 0.02 ^a^	0.46 ± 0.01 ^b^	0.18 ± 0.01 ^c^	0.61 ± 0.01 ^b^	0.12 ± 0.01 ^c^	<0.001
Phe + Tyr	1.31 ± 0.03 ^a^	0.64 ± 0.01 ^c^	0.25 ± 0.03 ^d^	0.93 ± 0.02 ^b^	0.44 ± 0.01 ^e^	<0.001
Lys	0.66 ± 0.01 ^a^	0.33 ± 0.01 ^b^	0.31 ± 0.01 ^b^	0.33 ± 0.01 ^b^	0.18 ± 0.01 ^c^	<0.001
Total	0.66 ± 0.01 ^a^	0.35 ± 0.00 ^c^	0.20 ± 0.01 ^d^	0.44 ± 0.00 ^b^	0.18 ± 0.01 ^d^	<0.001

Note: Values are mean ± SD (*n* = 6). Within a row, different lowercase superscripts indicate significant differences among ingredients (*p* < 0.05, Tukey HSD). “Total AAS” = (Total EAA mg/g)/345. One-way ANOVA across ingredients was performed within each row; *p* reported as three decimals or “<0.001”. AAS is presented as the raw ratio (not truncated at 1.00); if the journal prefers truncation at 1.00, we can provide an alternative capped table.

**Table 9:** The relative nutritional value equivalence of soybean meal at different time points.

MEQ(%).	t/min	Soybean Meal	Rapeseed Meal	Corn DDGS	Corn Gluten Meal	Corn Germ Meal	*p*-Value
Gastric digestion (2 h)	0	100.00 ^a^	32.17 ± 1.55 ^b^	6.69 ± 0.38 ^c^	15.62 ± 0.79 ^bc^	32.72 ± 1.61 ^b^	<0.001
	30	100.00 ^a^	30.22 ± 1.41 ^b^	2.71 ± 0.15 ^c^	5.38 ± 0.25 ^c^	12.79 ± 0.67 ^bc^	<0.001
	60	100.00 ^a^	22.04 ± 1.09 ^b^	2.76 ± 0.16 ^c^	4.17 ± 0.22 ^c^	16.70 ± 0.83 ^b^	<0.001
	90	100.00 ^a^	21.92 ± 1.08 ^b^	6.37 ± 0.36 ^c^	4.70 ± 0.25 ^c^	20.36 ± 1.02 ^b^	<0.001
	120	100.00 ^a^	20.20 ± 0.99 ^b^	53.35 ± 2.42 ^ab^	4.86 ± 0.26 ^c^	22.95 ± 1.14 ^b^	<0.001
Intestinal digestion (4 h)	0	100.00 ^a^	63.64 ± 3.02 ^b^	23.23 ± 1.12 ^c^	32.50 ± 1.64 ^c^	23.11 ± 1.11 ^c^	<0.001
	30	100.00 ^a^	94.52 ± 4.49 ^a^	34.92 ± 1.71 ^b^	58.80 ± 2.84 ^b^	20.83 ± 1.01 ^c^	<0.001
	60	100.00 ^a^	99.45 ± 0.36 ^a^	39.78 ± 3.13 ^b^	59.02 ± 3.62 ^b^	20.88 ± 2.14 ^c^	<0.001
	120	100.00 ^a^	97.44 ± 2.66 ^a^	45.49 ± 2.03 ^b^	62.87 ± 2.57 ^b^	23.00 ± 2.11 ^c^	<0.001
	180	100.00 ^a^	75.76 ± 3.79 ^b^	47.58 ± 2.37 ^c^	62.48 ± 3.12 ^b^	19.00 ± 0.95 ^c^	<0.001
	240	100.00 ^a^	29.52 ± 1.48 ^c^	11.10 ± 0.56 ^d^	35.30 ± 1.76 ^b^	7.82 ± 0.39 ^d^	<0.001

Note: Values are mean ± SD (*n* = 6). Within each row (fixed time point), different lowercase superscripts indicate significant differences among ingredients (*p* < 0.05, Tukey HSD). *p*-value represents one-way ANOVA results for ingredient effects at each time point (three decimals or “<0.001”).

**Table 10:** Protein structure and anti-nutritional factors affect the digestion kinetics of different plant protein sources.

Protein Source	Structural Traits	Anti-Nutritional Factors	Processing Improvement	Characteristics During Digestion
Soybean meal	mainly 7S/11S globulins; compact tertiary structure	Trypsin inhibitors (low), residual oligosaccharides	Starch removal; disulfide bond reduction; extrusion or fermentation	Fast gastric hydrolysis; high intestinal degree of hydrolysis (DH)
Rapeseed meal	High fiber, presence of glucosinolates; globulin- and albumin-rich	Trypsin inhibitors, phenolic compounds, glucosinolates	Heat treatment; enzymatic or microbial pre-treatment to degrade ANFs	Rapid gastric digestion, but intestinal digestion inhibited due to phenolics
Corn gluten meal	High zein content, strong disulfide cross-linking network	Low, but limited by hydrophobic protein matrix	Fiber reduction, enzymatic hydrolysis, microbial fermentation	Weak gastric digestion, strong intestinal digestion after pepsin exposure
Corn DDGS	Heat-damaged proteins, lipid–protein complex formation	Possible Maillard reaction products	Protein blending; mild enzymatic hydrolysis; solvent extraction	Low gastric and intestinal digestibility due to crosslinking
Corn germ meal	High fiber, low crude protein (CP), unbalanced amino acids	Non-starch polysaccharides (NSP), phytates	Fine grinding; carbohydrase supplementation; dehulling	Poor overall digestion; limited enzyme accessibility

## Data Availability

The data that support the findings of this study are not openly available due to reasons of sensitivity and are available from the corresponding author upon reasonable request.
